# Impact of *Enterobius vermicularis* infection and mebendazole treatment on intestinal microbiota and host immune response

**DOI:** 10.1371/journal.pntd.0005963

**Published:** 2017-09-25

**Authors:** Chin-An Yang, Chao Liang, Chia-Li Lin, Chiung-Tzu Hsiao, Ching-Tien Peng, Hung-Chih Lin, Jan-Gowth Chang

**Affiliations:** 1 Department of Laboratory Medicine, China Medical University Hospital, Taichung, Taiwan; 2 Division of General Pediatrics, Children’s Hospital of China Medical University, Taichung, Taiwan; 3 College of Medicine, China Medical University, Taichung, Taiwan; 4 Institute of Bioinformatics and Systems Biology, National Chiao Tung University, Hsinchu, Taiwan; 5 Skin Institute, China Medical University Hospital, Taichung, Taiwan; 6 Division of Pediatric Hematology-Oncology, Children's Hospital of China Medical University, Taichung, Taiwan; 7 Department of Biotechnology, Asia University, Taichung, Taiwan; 8 Division of Neonatology, Children’s Hospital and School of Chinese Medicine, China Medical University, Taichung, Taiwan; 9 Epigenome Research Center, China Medical University Hospital, Taichung, Taiwan; University of Washington School of Public Health, UNITED STATES

## Abstract

**Background:**

Previous studies on the association of enterobiasis and chronic inflammatory diseases have revealed contradictory results. The interaction of *Enterobius vermicularis* infection in particular with gut microbiota and induced immune responses has never been thoroughly examined.

**Methodology/Findings:**

In order to answer the question of whether exposure to pinworm and mebendazole can shift the intestinal microbial composition and immune responses, we recruited 109 (30 pinworm-negative, 79 pinworm-infected) first and fourth grade primary school children in Taichung, Taiwan, for a gut microbiome study and an intestinal cytokine and SIgA analysis. In the pinworm-infected individuals, fecal samples were collected again at 2 weeks after administration of 100 mg mebendazole. Gut microbiota diversity increased after *Enterobius* infection, and it peaked after administration of mebendazole. At the phylum level, pinworm infection and mebendazole deworming were associated with a decreased relative abundance of *Fusobacteria* and an increased proportion of *Actinobacteria*. At the genus level, the relative abundance of the probiotic *Bifidobacterium* increased after enterobiasis and mebendazole treatment. The intestinal SIgA level was found to be lower in the pinworm-infected group, and was elevated in half of the mebendazole-treated group. A higher proportion of pre-treatment *Salmonella spp*. was associated with a non-increase in SIgA after mebendazole deworming treatment.

**Conclusions/Significance:**

Childhood exposure to pinworm plus mebendazole is associated with increased bacterial diversity, an increased abundance of *Actinobacteria* including the probiotic *Bifidobacterium*, and a decreased proportion of *Fusobacteria*. The gut SIgA level was lower in the pinworm-infected group, and was increased in half of the individuals after mebendazole deworming treatment.

## Author summary

Whether human pinworm infection plus mebendazole deworming treatment can shift intestinal microbiota to a composition that is beneficial to the host and influence their mucosal immune response is currently unclear. In a cohort of 109 primary school children, we discovered that *Enterobius vermicularis* infection is associated with increased intestinal microbial diversity, a lowered relative abundance of *Fusobacteria* and an enriched proportion of *Actinobacteria*, including the probiotic *Bifidobacterium*. Mebendazole deworming was found to be correlated with a further increase in bacterial diversity. However, lower gut SIgA levels were detected in the pinworm infected group, and they were increased in only half of the subjects after mebendazole treatment.

## Introduction

The inverse epidemiology data of parasitosis and autoimmune diseases has led to the hypothesis that childhood exposure to parasites might have protective effects against the development of allergies and autoimmunity [1]. The immunomodulatory roles of helminth have been well studied, and several helminth-derived components might regulate the immune system [2, 3]. *Enterobius vermicularis* (human pinworm) is the most common parasite encountered in developed countries, and it has been suggested as a good candidate for testing the link between the “hygiene hypothesis” and disease [4]. In Taiwan, enterobiasis is found in about 0.6–3% of primary school children [5, 6]. A previous study using peri-anal tape tests and questionnaires with Taipei primary school children suggested a negative correlation between pinworm infection and allergic airway diseases [7]. Similarly, enterobiasis has been found to be associated with a decreased risk of allergic wheezing in Turkish school-aged children [8]. However, a large population cohort study collecting data of mebendazole prescriptions and chronic inflammatory diseases in Denmark showed that enterobiasis does not reduce the risk for asthma, type 1 diabetes (type 1 DM), arthritis, or inflammatory bowel disease (IBD) [9]. These abovementioned reports lacked mechanistic studies and did not examine the interaction between pinworm and intestinal microbiota.

The imbalance of pro-inflammatory and anti-inflammatory gut bacteria, or dysbiosis, is associated with autoimmune diseases such as type 1 DM, IBD, rheumatoid arthritis (RA), along with pro-inflammatory conditions, such as obesity, atherosclerosis and colon cancer [10–12]. Since the parasites and intestinal commensal bacteria reside in the same environment, interaction between these two micro-organisms is conceivable. In humans, it has been reported that helminth infections may increase intestinal bacterial diversity, and alter the composition of microbiota [13–15]. Gut microbiota is suspected as causing T helper type 1 (Th1) responses in *Trichuris muris* infections in mice, and *Schistosoma mansoni* has been suggested to cause Th1-mediated inflammation and granuloma formation via alteration of microbiota [16, 17]. Furthermore, the protective mucosal immune response against *Toxoplasma gondii* has been reported to be provided by gut microflora that stimulate dendritic cells [18]. Recently, Ramanan *et al*. reported that helminth infections may restore the number of goblet cells via suppression of an intestinal pro-inflammatory *Bacteroides* species, and thus protect genetically susceptible mice from the development of Crohn’s disease [19]. Therefore, the net immunomodulatory effect of pinworm on an individual may be dependent on its interaction with that individual’s intestinal microbiota.

In this study, we examined the impact of *Enterobius* exposure on the composition of gut microflora, and we investigated the interactions among pinworm, microbiota, and host immune responses in a prospective cohort of 109 primary school-aged children. Through our observations of differences in probiotic bacteria abundance and changes in gut levels of the protective secretory IgA (SIgA), we hypothesized the possible correlations of pinworm and mebendazole exposure with the inflammation status of the gut.

## Methods

### Ethics statement

The study cohort consisted of 109 primary school children (1^st^ and 4^th^ grades) who had undergone pinworm screening in 2015, Taichung, Taiwan. The study was approved by the Research Ethics Committee of China Medical University Hospital (CMUH104-REC1-115). Written informed consent was obtained from parents.

### Study design

A total of 30 children were tested negative for enterobiasis, while 79 were tested positive by anal tape screening. In children with positive pinworm anal tape results, additional stool samples were collected in tipped ova concentration tubes and were stained and fixed with freshly prepared merthiolate-iodine formaldehyde (MIF). After further mixing with ethyl acetate and centrifugation at 1500 rpm for 5 minutes, the sediments were examined carefully under light microscope to detect the presence of co-infected helminth eggs or protozoans as described previously [20]. Stool specimens were collected again from 65 pinworm-infected individuals 2 weeks following 100 mg mebendazole treatment, which underwent MIF-microscopic examination and 16s rRNA gene sequencing. We did not detect co-infection with other helminths or protozoans by MIF-concentration-sedimentation method in pinworm (+) samples before and after mebendazole treatment (Table 1). As shown in the flow diagram (S1 Fig), metagenomics analysis was performed on 30 pinworm (-), 65 paired pinworm (+) mebendazole (-) and pinworm (+) mebendazole (+) samples.

**Table 1:** Characteristics of the participants.

	Pinworm (-)	Pinworm (+)	Pinworm (+)	P value
		Mebendazole (-)	Mebendazole (+)	
Total number	30	79	65	NA
1^st^ Grade	15 (50.00%)	45 (56.96%)	40 (61.54%)	0.403*
4^th^ Grade	15 (50.00%)	34 (43.04%)	25 (38.46%)	
Female	17 (56.67%)	37 (46.84%)	32 (49.23%)	0.650*
Male	13 (43.33%)	42 (53.16%)	33 (50.77%)	
MIF concentration examination	NA	Pinworm egg: 1 (1.26%)	Pinworm egg: 0	NA
		Ova of other parasites: 0	Ova of other parasites: 0	
		Protozoans: 0	Protozoans: 0	
Mostly-meat diet	7 (23.33%)	NA	NA	0.537^#^/
				0.933^$^
Recent gastroenteritis	5 (16.67%)	NA	NA	0.378^#^/
				0.583^$^
Recent respiratory tract infection with oral medication	10 (33.33%) (8 had possible antibiotics usage)	NA	NA	0.014^#^/
				0.035^$^
Recent confirmed use of antibiotics	0	NA	NA	NA

*P values were calculated by Chi-square tests. In the pinworm (-) group, alpha and beta-diversity analyses were performed comparing gut microbiome composition of children with and without confounding factors.

^#^: alpha diversity p values;

^$^: beta diversity p values.

### DNA extraction and gene sequencing

Stool specimens were collected at home and transported to our laboratory within 3 hours in ice, and were fixed in Transwab tubes (Sigma, Dorset, UK). DNA extraction was performed using the QIAamp DNA Stool Mini Kit (Qiagen, California, USA). PCR primers F515 (5’-GTGCCAGCMGCCGCGGTAA-3’) and R806 (5’-GGACTACHVGGGTWTCTAAT-3’), were designed to amplify the V4 domain of bacterial 16S ribosomal RNA gene as described previously [21]. The Nextera adapter sequence (Illumina, California, USA) was added to the 5’-end of the primer set for library preparation. PCR using 50~150 ng DNA was performed with 1 cycle of 98°C for 30 sec, 30 cycles of 98°C for 10 sec, 60°C for 30 sec, 72°C for 30 sec, and a final extension of 72°C for 5 min. Amplicons were purified using the AMPure XP Beads (Beckman Coulter, Indianapolis, USA), and quantified using Nanophotometer (IMPLEN, München, Germany). The Illumina Nextera Index Primer kit was used to create the library. The qualities and quantities of purified libraries were checked by 2% agarose gel electrophoresis, Qubit (Thermo Fisher Scientific, Massachusetts, USA) and qPCR methods. Finally, libraries were normalized to the same concentration and sequenced by Illumina Miseq sequencer.

### Bioinformatic analysis of 16S rRNA gene sequencing data

FASTX-Toolkit (http://hannonlab.cshl.edu/fastx_toolkit) was used to process the raw read data files. Sequence qualities were checked in 3 steps: (i) The minimal acceptable Phred quality score of sequences was 20 (having over 70% of the sequence bases ≥ 20). (ii) Following quality trimming from the sequence tail, the sequences over 100 bp and those with an acceptable Phred quality score of 20 were retained. (iii) Both forward and reverse sequencing reads which met the first and second requirements were retained for subsequent analyses.

UPARSE [22] was used to create operational taxonomic unit (OTU) clustering. Bowtie2 [23] was then used to align OTUs with 16S rRNA gene sequences of bacteria. These sequences were taken from the SILVA ribosomal RNA sequence database (version 115). Following sequence data collection, sequences were extracted by V4 forward primer and reverse primer. To prevent repetitive sequence assignments, V4 sequences from SILVA were then grouped into several clusters by 97% similarity using UCLUST. A standard of 97% similarity with the database was applied.

### Intestinal SIgA and cytokine detection

Fecal samples were weighed before adding equal amounts of sterile PBS together with Pierce proteinase inhibitor (Thermo Fisher Scientific). After thorough mixing and centrifugation at 10000 g for 10 minutes, the fecal supernatants were stored at -80°C until analysis. Stool secretory IgA (SIgA) was analyzed using Immundiagnostik ELISA kit (Bensheim, Germany), IL-1ß, and IL-4 levels were measured using Quantikine ELISA kits (R&D Systems, Minneapolis, USA) according to manufacturer’s instructions.

We further grouped the samples according to levels of SIgA, IL-1ß, and IL-4. Based on median levels detected, SIgA was considered high at >150 μg/ml, and low at <80 μg/ml; IL-1ß was high at >10 pg/ml, and low at <0.5 pg/ml; and IL-4 was high at >10 pg/ml, and low at <5 pg/ml. In paired samples (collected before and after mebendazole treatment), SIgA level was considered to be elevated given a greater than 1.1-fold increase.

### Statistical analysis

A rarefaction process was applied to normalize the operational taxonomic unit (OTU) table following taxonomy assignment in the bioinformatic analyses. Alpha diversity (Shannon index, inverse Simpson index and richness) was calculated. Beta diversity using weighted UniFrac distance metrics [24], principal coordinate analysis (PCoA) and unsupervised clustering were performed. Multiple response permutation procedure (MRPP) in an R package “vegan” (https://cran.r-project.org/web/package=vegan) was performed to assess community difference in PCoA. Wilcoxon rank sum test were used to compare non-paired variables (e.g., P- v.s. P+M-), and Wilcoxon signed rank test were used to compare paired variables (e.g., P+M- v.s. P+M+). The differentially expressed bacteria were filtered by the following criteria: (i) P value < 0.05 (ii) Fold change > 1.40 or < 0.71 (iii) At least one group achieved an average relative abundance of 0.5%. Sex and pair factors were adjusted. For multiple group comparisons the false discovery rate (FDR) was controlled by using Benjamini-Hochberg (BH) FDR multiple test correction. Pathway enrichment analysis was performed using an R package 'Tax4Fun' [25]. ANOVA test was used to calculate the enrichment difference. Mann-Whitney U tests using GraphPad Prism version 5 were performed to compare fecal cytokine and SIgA levels between groups. Wilcoxon signed rank test was used to analyse the paired stool SIgA data before and after mebendazole treatment.

## Results

### Study population and characteristics of intestinal microbiota

We analyzed the influence of pinworm infection on gut microbiome in a cohort of 109 children in the first or fourth grade of primary school. Grade and sex effects were both insignificant among the groups (p = 0.650, p = 0.403, respectively, Table 1). In the pinworm (+) group, additional stool specimens were collected to perform MIF concentration sedimentation procedure on to detect co-infection of other parasites. As shown in Table 1, no co-infection was detected. In the pinworm (-) group, possible confounding factors including a mostly-meat diet, recent (within these 2 months) gastroenteritis, recent respiratory tract infection with oral medication, and recent confirmed use of antibiotics were recorded, and diversity analysis of the gut microbiota showed that recent respiratory tract infection with oral medication might decrease the intestinal microbial diversity (Table 1). Further differential abundance analysis at the phylum level showed that children with recent respiratory tract infection and oral medication (8 of 10 had possible antibiotics usage) had a trend to correlate with relatively less abundance of *Fusobacteria* (0.01%± 0.18% vs. 1.65%±4.62%, p = 0.03, FDR = 0.17). Information about the above confounding factors was not available in the pinworm (+) group.

**Fig 1:**
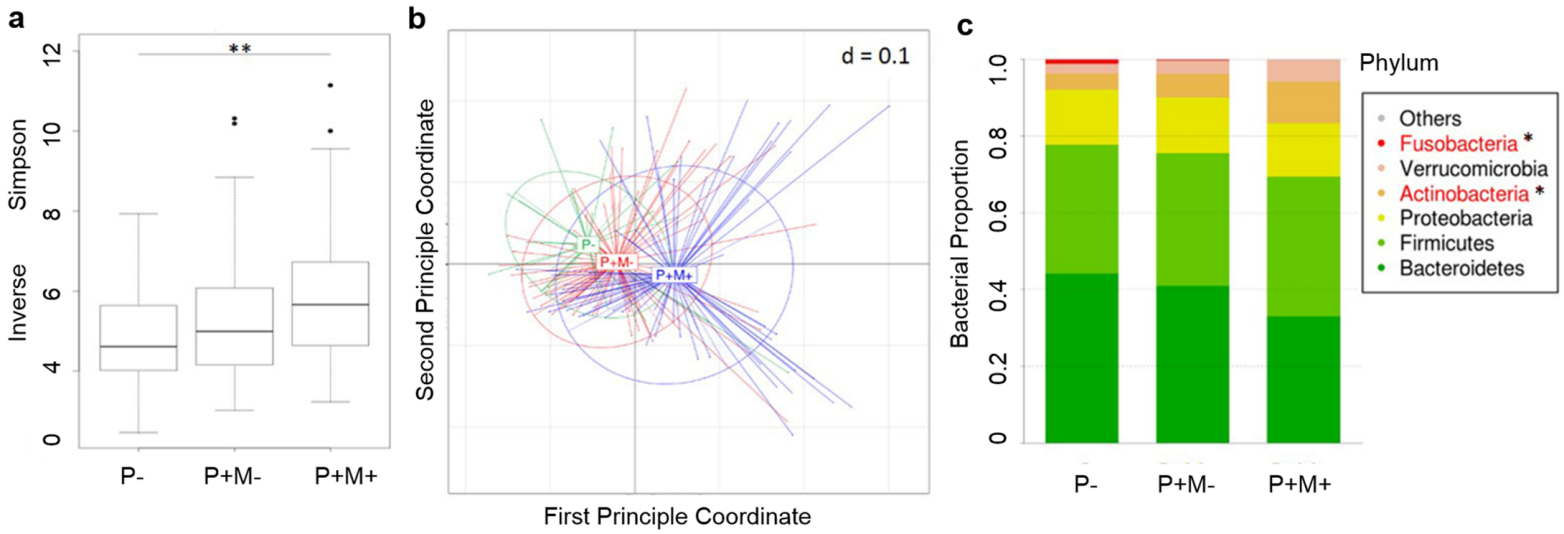
Intestinal microbiota community comparison of pinworm infection and mebendazole treatment groups. **a**. Boxplot of alpha diversity (Inverse Simpson index) is shown in genus level. **: p<0.01 by ANOVA test. P-: pinworm negative, n = 30; P+M-: pinworm positive before mebendazole treatment, n = 65; P+M+: pinworm positive after mebendazole treatment, n = 65. **b**. Principal coordinate analysis of weighted UniFrac beta diversity between subjects, colored by community subgroups. **c**. Bacterial relative abundance distribution of the community subgroups and differentially expressed phyla. *: p < 0.05 by Kruskal-Wallis test.

The alpha diversity of the pinworm (+) mebendazole (+) group was significantly higher than the pinworm (-) group (inverse Simpson index, p = 0.002), and the alpha diversity was only marginally higher when comparing the microbial composition between the pinworm (+) mebendazole (-) group and the pinworm (-) group (p = 0.061, Fig 1a). The principle coordinate analysis also showed a significant beta diversity difference among the 3 groups (pinworm (+) mebendazole (+) group vs. pinworm (-) group, p = 0.001, Fig 1b).

Analysis of the intestinal microbiome operational taxonomic units (OTUs) of our cohort revealed the following major bacterial phyla: *Bacteroidetes*, *Firmicutes*, *Proteobacteria*, *Actinobacteria*, *Verrucobacteria*, and *Fusobacteria*. The phylum microbial distribution pattern differed significantly in the proportion of *Actinobacteria* (pinworm (+) mebendazole (+) vs. pinworm (-), 1.08% ± 1.15% vs. 4.23% ± 5.52%, fold = 2.56, P = 5.19 x 10^−4^, FDR = 0.012, Fig 1c) and *Fusobacteria* (pinworm (+) mebendazole (+) vs. pinworm (-), 0.04% ± 0.17% vs. 1.10% ± 3.82%, fold = 0.04, P = 3.30 x 10^−3^, FDR = 0.012, Fig 1c).

### Impact of pinworm infection and mebendazole deworming treatment on intestinal microbial composition

**Fig 2:**
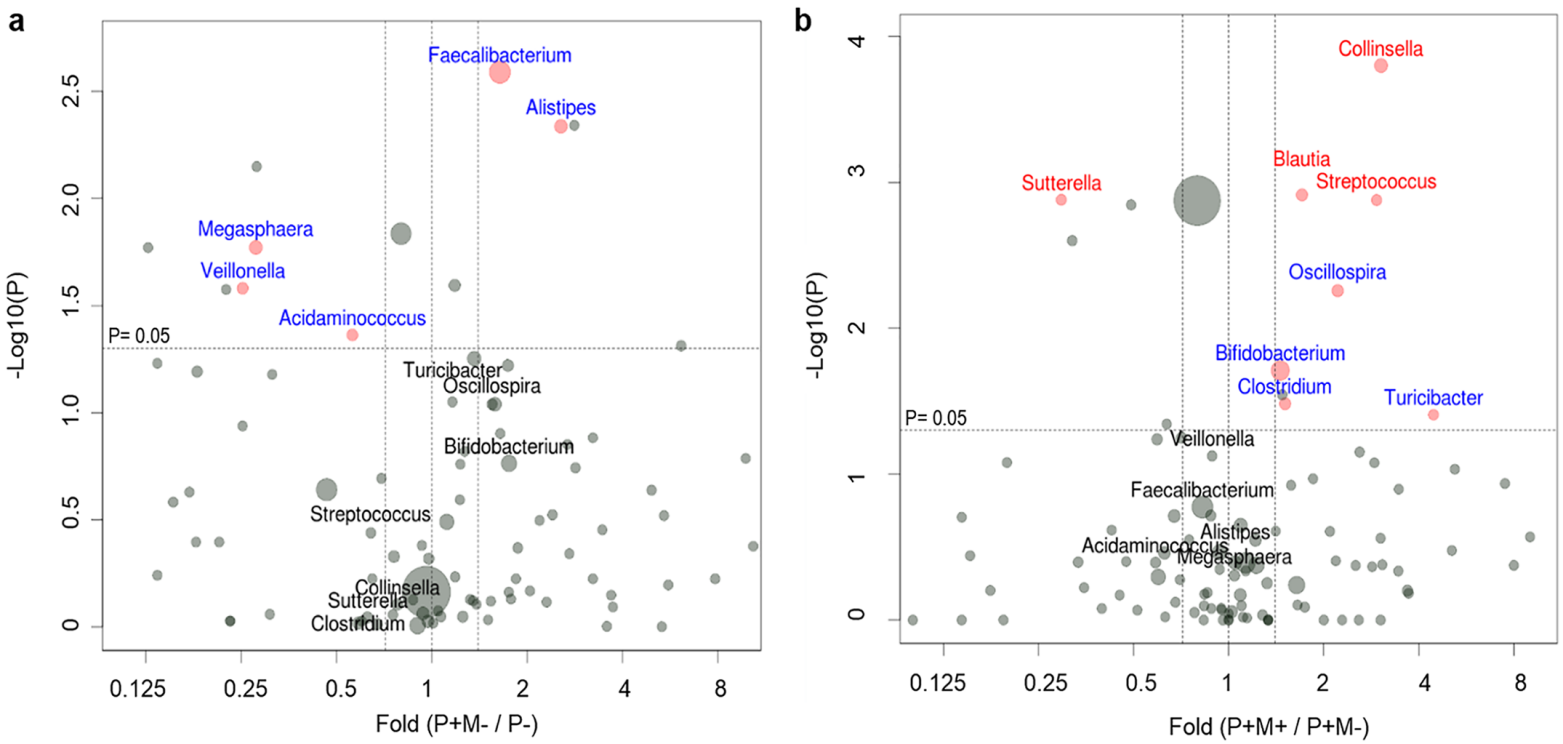
Visualization of differentially expressed genus-level bacteria by volcano plots. Red dots represent significant taxa analyzed by Wilcoxon rank sum test (pinworm (+) mebendazole (-) vs. pinworm (-)) and Wilcoxon signed rank test (paired pinworm (+) mebendazole (+) vs. pinworm (+) mebendazole (-)). After p value corrections, those taxa remained to be significant (FDR< 0.05) are written in red, and bacterial species with FDR> 0.05 are written in blue. Boundaries of significant fold change and p value of Wilcoxon tests are shown in dashed lines. Dot size represents average relative abundance. **a**. Impact of pinworm infection on gut microbiome. Comparison was made between the P+M- group and the P- group. **b**. Impact of mebendazole administration on gut microbiome. Comparison was made between P+M+ group and P+M- group. P-: pinworm negative, n = 30; P+M-: pinworm positive before mebendazole treatment, n = 65; P+M+: pinworm positive after mebendazole treatment, n = 65.

At the genus level, a trend of higher relative abundance of *Alistipes* (fold = 2.56, p = 0.008) and *Faecalibacterium* (fold = 1.64, p = 0.004), and a decreased proportion of *Fusobacterium* (fold = 0.18, P = 0.050), *Veilonella* (fold = 0.25, p = 0.042), *Megasphaera* (fold = 0.28, p = 0.021), and *Acidaminococcus* (fold = 0.56, p = 0.030) were found in the pinworm (+) mebendazole (-) group as compared with the pinworm (-) group (Fig 2a). However, the corrected p values (FDRs) for all differentially distributed taxa were all > 0.05. In the 65 pinworm (+) mebendazole (+) subjects, the intestinal bacterial diversity further increased and was correlated with significantly more abundant *Collinsella* (fold = 3.04, p = 1.58 x 10^−4^, FDR = 0.028), *Streptococcus* (fold = 2.94, p = 1.32 x 10^−3^, FDR = 0.043), *Blautia* (fold = 1.71, p = 1.22 x 10^−3^, FDR = 0.043), as well as a lower proportion of *Suterrella* (fold = 0.30, p = 1.32 x 10^−3^, FDR = 0.043), as compared with the microbial composition of pinworm (+) mebendazole (-) group, Fig 2b. The relative abundance of the probiotic *Bifidobacterium* increased after pinworm infection, and it became even higher in the mebendazole treated group (pinworm (+) mebendazole (+) vs. pinworm (-), 7.32% ± 9.28% vs. 2.86% ± 3.67%, p = 1.97 x 10–3, FDR = 0.100, Fig 2a and 2b).

**Fig 3:**
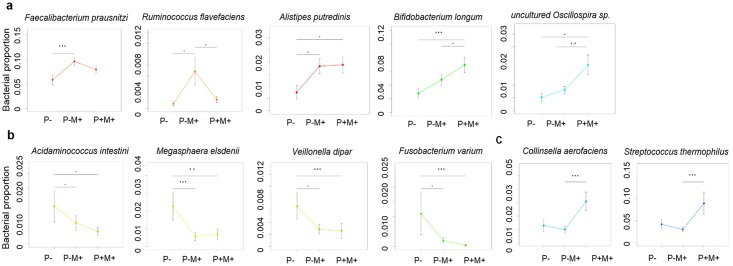
Changes in proportions of intestinal bacterial species after pinworm infection or mebendazole deworming. **a**. Bacterial species increased after pinworm infection. **b**. Bacterial species decreased after pinworm infection. **c**. Bacterial species increased after mebendazole deworming. ***: p <0.001, **: p <0.01, *: p<0.05 by Wilcoxon rank sum test (P- vs. P+M-, P- vs. P+M+) or Wilcoxon signed rank test (P+M- vs. P+M+, paired). Bars represent mean±SEM.

At the species level, pinworm infection was associated with a trend of increased proportions of *Faecalibacterium prausnitzii*, *Ruminococcus flavefaciens*, *Alistipes purtredinis*, *Bifidobacterium longum* and *uncultured Oscillospira sp*. (Fig 3a, percentages and p values are shown in S1 Table), as well as a trend of decreased relative abundance of *Acidaminococcus intestine*, *Megasphaera elsdenii*, *Veillonella dispar* and *Fusobacterium varium* (Fig 3b and S1 Table). The relative abundance of *Faecalibacterium prausnitzii* and *Ruminococcus flavefaciens* were lower, while the proportion of *Bifidobacterium longum* and *uncultured Oscillospira sp*. were higher after mebendazole deworming (Fig 3a, S1 Table). Mebendazole deworming was not associated with an increase in the relative abundance of *Acidaminococcus intestine*, *Megasphaera elsdenii*, *Veillonella dispar* and *Fusobacterium varium* as compared with the pinworm (+) mebedazole (-) group (Fig 3b).

Furthermore, as shown in Fig 3c, the relative abundance of *Collinsella aerofaciens* and *Streptococcus thermophilus* did not change significantly after pinworm infection; however, an increase in the proportion of these 2 species was detected 2 weeks after mebendazole deworming treatment (*Collinsella aerofaciens*: pinworm (+) mebendazole (+) vs. pinworm (+) mebendazole (-), 3.07% ± 5.52% vs. 1.00% ± 2.00%, fold = 3.08, p = 9.18 x 10^−5^, FDR = 0.034; *Streptococcus thermophiles*: pinworm (+) mebendazole (+) vs. pinworm (+) mebendazole (-), 0.89% ± 2.04% vs. 0.31% ± 0.42%, fold = 2.93, p = 0.003, FDR = 0.158, Fig 3c).

### Enrichment of microbiome by pinworm infection and mebendazole treatment

Taxonomic annotation-based enrichment analysis showed that the abundance of Gram-positive and endospore-forming bacteria was increased in the pinworm (+) mebendazole (+) group, as compared with the pinworm (-) group (p = 0.0001 and p = 0.001, respectively, S2a and S2b Fig). Further pathway enrichment analysis suggested that an enriched gut microbiome involving fat absorption and digestion pathway (ko04975) was associated with pinworm infection (pinworm (+) mebendazole (-) vs. pinworm (-), fold = 2.49, p = 0.014, S2c Fig), and the statistical significance remained when comparing the microbiota of the mebendazole treated group with the pinworm uninfected group (fold = 2.54, p = 0.007, S2c Fig). In addition, exposure to pinworm and mebendazole was found to be correlated with the enrichment of the gut microbiome involving the fatty acid elongation pathway (ko00062, pinworm (+) mebendazole (+) vs. pinworm (-), fold = 2.01, p = 0.001, S2d Fig) and the caffeine metabolism pathway (ko00232, pinworm (+) mebendazole (+) vs. pinworm (-), fold = 2.04, p = 0.002, S2e Fig).

### Association of fecal SIgA and cytokine levels with pinworm infection and intestinal bacterial abundance

**Fig 4:**
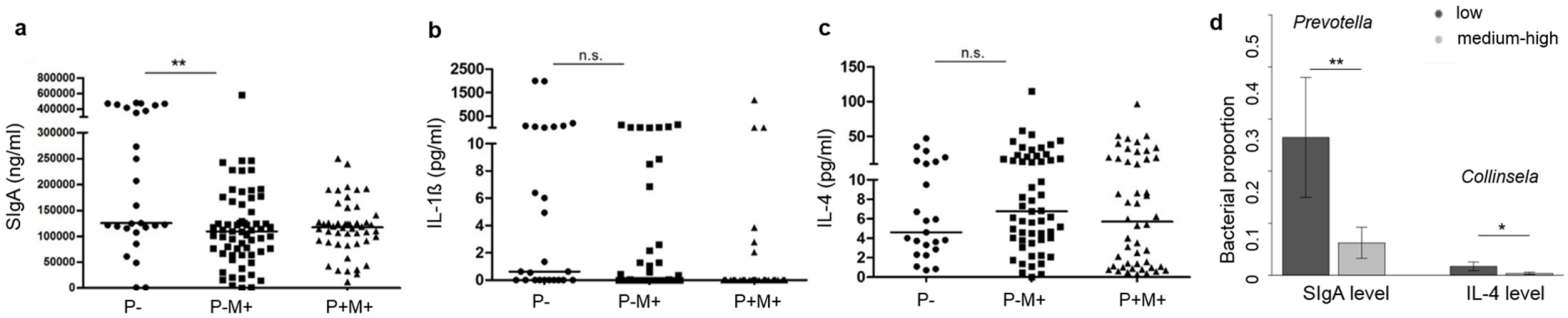
Associations of SIgA/ cytokine levels with pinworm infection and with relative abundance of gut bacterial species. **a**. Fecal SIgA, **b**. fecal IL-1ß, and **c**. fecal IL-4 levels in P-, P+M-, and P+M+ groups. **: p<0.01 by Mann-Whitney U test. Lines represent medians. **d**. Correlation of the relative abundance of *Prevotella* with gut SIgA levels, and correlation of the relative abundance of *Collinsella* in the P- group (low SIgA: <80000 ng/ml, n = 5; medium-high SIgA: ≥ 150000 ng/ml, n = 22; low IL-4: < 5 pg/ml, n = 12, medium-high: ≥5, n = 11). Bars represent mean±SEM. *: p<0.05, **: p<0.01 by Wilcoxon rank sum test, corrected for the following factors: recent gastroenteritis and recent respiratory tract infection with oral medication.

To analyze the impact of *Enterobius* exposure on a host’s intestinal immune response, stool samples from pinworm-uninfected, pinworm-infected and untreated, and pinworm-infected and treated groups were collected and measured for their SIgA, IL-1ß and IL-4 levels. Pinworm infection was found to be associated with a significant decrease in gut SIgA levels (median level of uninfected vs. pinworm (+) mebendazole (-) group: 125.59 μg/ml vs. 109.56 μg/ml, p<0.01, Fig 4a). The amount of fecal IL-1ß and IL-4 were similar before and after pinworm infection (Fig 4b and 4c).

Furthermore, the fecal levels of SIgA and cytokines were grouped into low, medium and high as described in the Methods Section. In the pinworm (-) group, possible confounding factors such as recent gastroenteritis and respiratory tract infection with oral medication were collected; and in this group, correlation studies of SIgA and cytokine levels with intestinal microbial taxa revealed an association of a higher *Prevotella* proportion with a decreased amount of gut SIgA (relative abundance of *Prevotella* in SIgA medium-high vs. SIgA low, 6.22% ± 2.97% vs. 26.46% ± 11.52%, p = 0.006 (corrected for respiratory and gastrointestinal infection factors), Fig 4d), and association of a higher *Collinsella* abundance with a decreased amount of gut IL-4 (relative abundance of *Collinsella* in IL-4 medium-high vs. IL-4 low, 0.36% ± 0.21% vs. 1.69% ± 0.84%, p = 0.043 (corrected for respiratory and gastrointestinal infection factors), Fig 4d).

### Microbiota associated with increased SIgA level after mebendazole treatment

**Fig 5:**
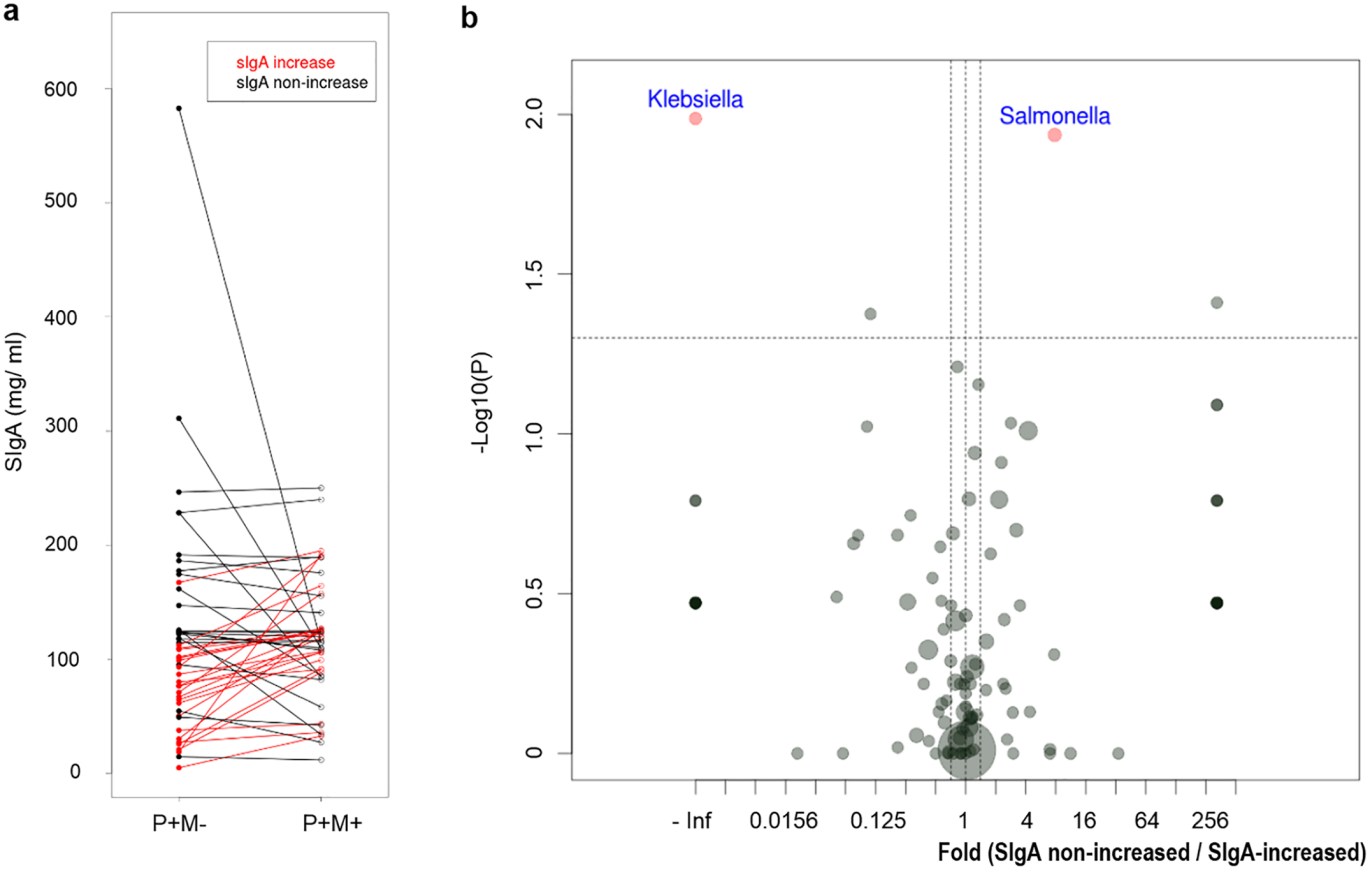
Gut microbial composition differences in SIgA-increased and SIgA-non-increased groups. **a**. 48 pinworm (+) mebendazole (-) and pinworm (+) mebendazole (+) paired fecal samples were evaluated for SIgA levels. 24 subjects showed >1.1 fold increase of SIgA after mebendazole deworming (SIgA-increased group, marked in red), and the other 24 subjects fell in the SIgA-non-increased group (marked in black). **b**. Volcano plot showing differentially expressed genus-level bacteria comparing the untreated, pinworm-infected P+M- samples of SIgA non-increased group and the P+M- samples of SIgA-increased group. Significant microbes analyzed by Wilcoxon rank sum tests are shown in red dots. Boundaries of significant fold change and p value are shown in dashed lines. Dot size represents average relative abundance.

**Fig 6:**
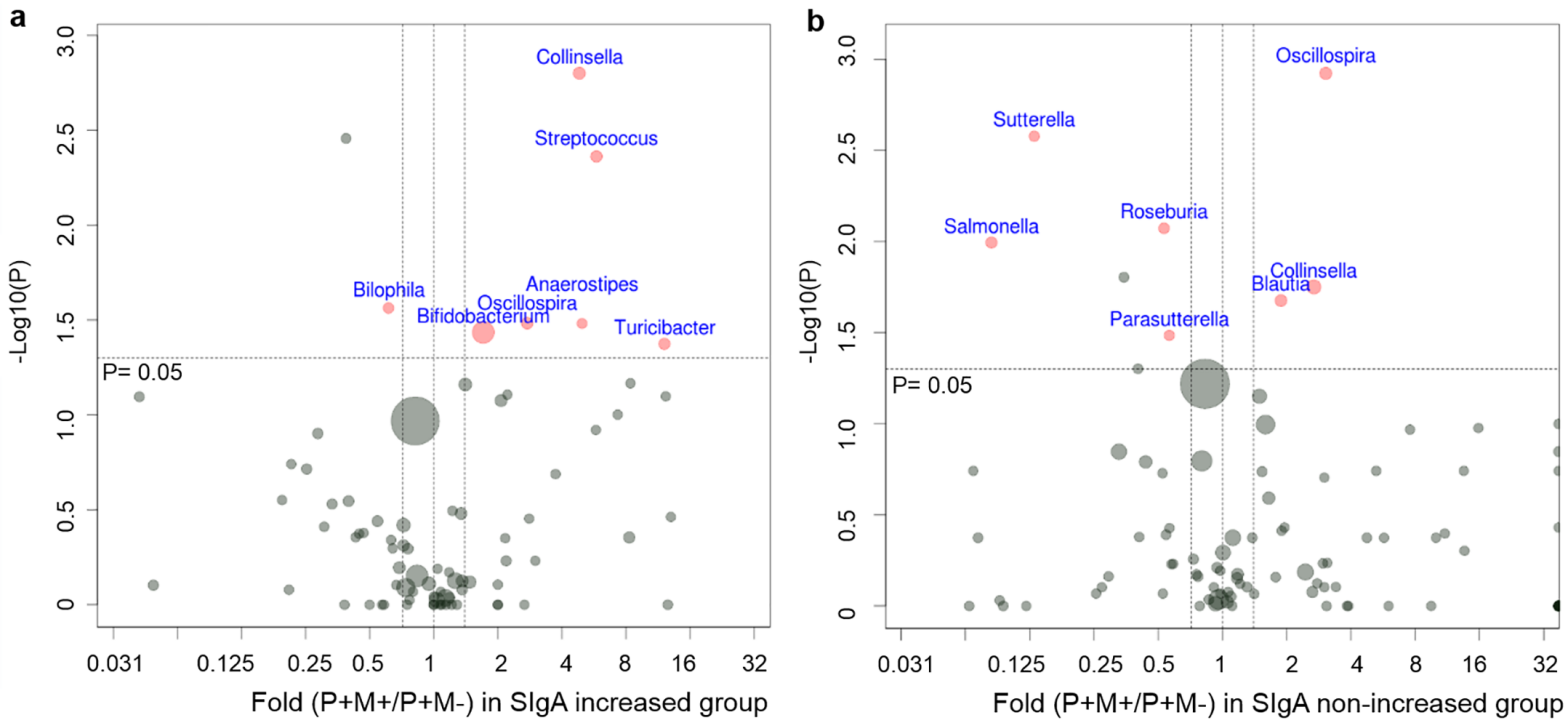
The shifts of intestinal microbiota after mebendazole deworming in the SIgA-increased (a) and SIgA non-increased groups (b). Differentially expressed bacterial genera comparing P+M+ samples vs. P+M- samples are shown in volcano plots.

After mebendazole deworming, the amount of intestinal SIgA only increased in half of the treated subjects (Fig 5a). We then investigated the fecal microbial composition of the mebendazole-treated samples with and without SIgA-restoration. Before mebendazole deworming, the SIgA-non-increased group was associated with a higher proportion of the gut pathogen *Salmonella* (SIgA-non-increased vs. SIgA-increased group, 1.40% ± 3.21% vs. 0.18% ± 0.45%, p = 0.012, Fig 5b), and a lower abundance of the commensal *Klebsiella*, as compared with the SIgA-increased group (0.00% ± 0.00% vs. 0.74% ± 2.84%, p = 0.010, Fig 5b). Furthermore, mebendazole deworming was associated with increased percentages of *Bifidobacterium* and *Streptococcus* in the SIgA-increased specimens (P+M+ vs. P+M-, *Bifidobacterium*: 9.96% ± 12.53% vs. 5.83% ± 8.47%, p = 0.037; *Streptococcus*: 1.58% ± 3.23% vs. 0.27% ± 0.35%, p = 0.004, Fig 6a), and a decreased relative abundance of *Salmonella* in the SIgA non-increased subjects (P+M+ vs. P+M-, 0.12% ± 0.48% vs. 1.40% ± 3.21%, p = 0.010, Fig 6b). A mebendazole-deworming associated increase in the proportions of *Collinsella* was observed in both the SIgA-increased and the SIgA-non-increased groups (P+M+ vs. P+M-, SIgA-increased group: 2.08% ± 3.43% vs. 0.43% ± 0.69%, p = 0.002; SIgA-non-increased group: 3.71% ± 5.95% vs. 1.38% ± 2.43%, p = 0.018, Fig 6a and 6b).

At the species level, a higher proportion of *Salmonella enterica* was noted in pre-treatment samples of the SIgA-non-increased group (fold = 7.81, p = 0.012), and it decreased after mebendazole deworming (fold = 0.08, p = 0.010) (S3a and S3b Fig). The association of the increased relative abundance of the probiotic bacteria *Streptococcus thermophilus* and *Bifidobacterium longum* with mebendazole deworming was detected only in the SIgA-increased group (*Streptococcus thermophilus*: fold = 5.84, p = 0.006; *Bifidobacterium longum*: fold = 1.67, p = 0.046, S3c Fig).

## Discussion

We examined the impact of *Enterobius vermicularis* infection and the effect of mebendazole deworming on intestinal microbial composition and mucosal immune responses in 109 primary school children. Both enterobiasis and mebendazole deworming were associated with altered intestinal microbiome.

Consistent with a previous study on helminth-infected microbiota [14], pinworm infection in our study was associated with increased intestinal bacterial diversity. Furthermore, it has been reported that hookworm infection in human subjects with celiac disease could not only increase gut microbial richness, but also regulate gluten-induced inflammation [26]. We did not conduct a pinworm infection trial on human with chronic inflammatory diseases. However, in our study we found that *Enterobiasis* was associated with an increased relative abundance of *Faecalibacterium prausnitzii* and *Alistipes* species. *Faecalibacterium prausnitzii* has been shown to be underrepresented in the gut of patients with IBD, type 2 DM, and obesity; while *Alistipes spp*. has been reported to be overrepresented in irritable bowel syndrome (IBS) patients reporting abdominal pain, and in depressive individuals, suggesting a possible role in disturbing the intestinal serotonergic system [10, 27–31]. The pro-inflammatory role of *Alistipes spp*. remains speculative. As for the bacterial taxa which showed lower percentages after pinworm infection, both *Veillonella spp*. and *Fusobacterium spp*. have been suggested to be correlated with pro-inflammatory conditions such as ulcerative colitis and colon cancer, and *Veillonella dispar* and *Fusobacterium varium* have been detected in colon carcinoma in adenoma [32–34]. Whether bacterial species altered by pinworm favors an anti-inflammatory profile requires further investigations.

Anthony *et al*. (2007) noted that the major immune response raised against helminth infection is the Th2-type response, consisting of an expansion of Th2 helper T cells, eosinophils, mast cells, basophils, elevated IgE, IL-4 and other cytokines, including IL-5 and IL-13 [2]. A previous study in children in central Greece suggested a Th2-type oriented response to pinworms based on elevated serum levels of IgE and eosinophil cationic protein (ECP) [35]. However, we found no differences in the fecal IL-4 levels among the pinworm (-), pinworm (+) mebendazole (-), and pinworm (+) mebendazole (+) groups. In our study, mebendazole deworming was found to be associated with an increased proportion of *Collinsella*. Interestingly, in the pinworm (-) group, after correction for possible confounding factors including recent gastroenteritis and respiratory infection with oral medication (and possible antibiotics usage), an inverse correlation between *Collinsella* abundance and gut IL-4 level was detected. Additional research on germ-free animals is needed to evaluate the effect of pinworm and mebendazole on gut *Collinsella* and IL-4 levels.

IL-1ß is another cytokine that could be altered by parasites, and its over-activation is associated with chronic inflammatory diseases [36]. To establish the chronicity of infection, the murine helminth *Heligmosomoides polygyrus bakeri* (Hp) has been observed to downregulate the host’s IL-4 response by promoting IL-1ß production [37]. In contrast, the parasite *Fasciola hepatica* has been seen to directly inhibit host IL-1ß secretion [38]. We found that pinworm infection alone did not significantly alter the fecal IL-1ß.

The principle immunoglobulin involved in combating intestinal microbial infection and maintaining mucosal homeostasis with commensal bacteria is SIgA, which mediates anti-inflammatory functions via multiple mechanisms [39]. A lack of SIgA can also cause inflammatory diseases [40, 41]. *Bacteroides thetaiotaomicron* colonization in mice has been reported to elevate SIgA levels via an influx of IgA-producing B cells and an increase of polymeric immunoglobulin receptor (pIgR) that mediates the transport of IgA across epithelia [42]. In this study, pinworm infection was found to be correlated with lower gut SIgA level. Furthermore, the amount of intestinal SIgA was found to be negatively associated with the relative abundance of *Prevotella*. Whether this pinworm—microbial interaction influences gut SIgA production is unclear. Of note, after mebendazole deworming treatment, SIgA levels increased in half of the pinworm-infected subjects. We observed that the intestinal pathogen *Salmonella enterica* was overrepresented in the SIgA non-increased group, when compared with the SIgA-increased group. Although the relative proportion of *Salmonella enterica* was lower after mebendazole deworming in the SIgA-non-increased samples, the increase of the probiotic species *Streptococcus thermophilus* and *Bifidobacterium longum* following mebendazole treatment was only observed in the SIgA-increased group. Our results suggest that the relative abundance of *Salmonella* might have a negative effect on the mebendazole deworming -associated increase in the amount of SIgA and probiotic species in the gut.

Mebendazole is a classic anti-helminth drug, which is well-tolerated [43], and is routinely given to pinworm-positive school aged children. A Danish study on pinworm infection and risk of chronic inflammatory diseases even used mebendazole treatment history as a surrogate for enterobiasis diagnosis [9]. The results of our study show that increased percentages of the known probiotic species, *Streptococcus thermophilus*, and another anti-inflammatory bacterium, *Collinsella aerofaciens* [44], could be associated with mebendazole deworming, but not with pinworm infection alone. Our study is limited in that we did not use anal tape, a more sensitive method for detection of pinworm eggs than MIF concentration sedimentation, to evaluate the efficacy of mebendazole deworming. However, *Wang CC et al*. reported in *J Microbiol Immunol Infect*. 2009 that the efficacy of mebendazole treatment on eradicating pinworm in primary school children in Taichung, Taiwan, was 96% [6]. Mebendazole was found to have anti-inflammatory, anti-angiogenesis and oncogene-suppressing activities in a mouse model of colon cancer initiation [45]. The direct effect of mebendazole on gut microbiota composition remains to be investigated. An enrichment pathway analysis of the microbiome in our study showed that the increase in the percentages of microbes involved in the metabolism of fatty acid elongation and caffeine after pinworm infection only became significant when a comparison was made between the pinworm (+) mebendazole (+) group and the pinworm (-) group. Further metabolomics studies are needed to evaluate if pinworm and mebendazole treatment could alter the metabolism of commensal bacteria and subsequently influence a host’s immune system.

In our study, the change in microbial composition detected two weeks after administration of mebendazole on pinworm-infected children could be confounded by late onset effects of enterobiasis. A larger prospective cohort study with a longer follow-up on gut microbiomes will help to determine more exactly the duration and dynamics of the change in microbiota and in SIgA levels after mebendazole deworming treatment. Host genetics and diet are confounding factors for long-term follow up. Variations in the human genome have been found to favor the colonization of different gut microbiota [46]. A high fat and low fiber diet has been shown to be associated with reduced beneficial microbes producing short chain fatty acids (SCFAs) and thus such a diet increases the risk of inflammatory and autoimmune diseases [47]. In addition, our results revealed that recent respiratory tract infection with oral medication usage in the study population may be inversely correlated with intestinal microbial diversity and a decreased relative abundance of *Fusobacterium* and *Acidaminococcus*, which might interfere with the effect of pinworm infection. Thus, differential host genetics, lifestyles, respiratory tract infection rates and medication usage can all contribute to the inconsistent association of enterobiasis and the risk of inflammatory diseases observed in pediatric cohorts of various countries.

In conclusion, *Enterobius vermicularis* infections are associated with increased intestinal microbial diversity, and decreased gut SIgA levels. Several bacterial taxa exhibited differential abundance in pinworm (-), pinworm (+) mebendazole (-), and pinworm (+) mebendazole (+) groups. Mebendazole deworming was correlated with increased intestinal SIgA level and a higher proportion of probiotic bacteria in half of the infected subjects. To better understand the causal relationships of pinworm infection and mebendazole treatment on gut microbial composition and hosts’ immune responses, more experiments including animal studies are needed.

## Supporting information

S1 Table (DOCX)

S1 Fig Flow diagram. P(-): pinworm-negative; P(+)M(-): pinworm-infected, before mebendazole treatment; P(+)M(+): pinworm-infected, 2 weeks after mebendazole treatment.
(TIF)

S2 Fig Microbiology characteristics and pathway enrichment analysis. **A-B**. Box plots showing microbiology characteristic enrichment analyses based on Gram staining (**a**) and endospore-forming features (**b**). ***: p <0.001 by ANOVA test. **c-e**. Pathway enrichment analyses showing significant increase in fat metabolism (**c**), fatty acid elongation (**d**) and caffeine metabolism (**e**) pathways after pinworm infection plus mebendazole treatment. R package “Tax4Fun” was used to transform the OTU table into pathway activity value. ***: p <0.001, **: P<0.01, *: P<0.05 by ANOVA tests.
(TIF)

S3 Fig Gut bacterial species composition differences in SIgA-increased and SIgA-non-increased groups. Volcano plots showing differentially expressed species-level bacteria comparing the untreated, pinworm-infected P+M- samples of SIgA non-increased group vs. the P+M- samples of SIgA-increased group (**a**), P+M+ samples vs. P+M- samples in SIgA non-increased group (**b**), and P+M+ samples vs. P+M- samples in SIgA-increased group (**c**). Red dots represent significant taxa analyzed by Wilcoxon tests. After p value corrections, those taxa remained to be significant (FDR< 0.05) are written in red, bacterial species with FDR> 0.05 are written in blue. Boundaries of significant fold change and p value are shown in dashed lines. Dot size represents average relative abundance.
(TIF)
