# Supplementary material for: Impact of Enterobius vermicularis infection and mebendazole treatment on intestinal microbiota and host immune response
Source: PLoS Negl Trop Dis. 2017 Sep 25;11(9):e0005963. doi: 10.1371/journal.pntd.0005963 (PMC5629029; doi:10.1371/journal.pntd.0005963)
Supplement: S1 Table — (DOCX) [file pntd.0005963.s001.docx]

**Table S1.** Bacterial species with differential abundance among groups

| **Bacterial species** | **Proportion in P(-)**  **%** | **Proportion in P(+)M(-)**  **%** | **Proportion in P(+)M(+)**  **%** | **P value / FDR of**  **P(-) vs. P(+)M(-)** | **P value / FDR of**  **P(+)M(-) vs. P(+)M(+)** |
| --- | --- | --- | --- | --- | --- |
| *Faecalibacterium prausnitzii* | 5.83 ± 5.56 | 9.57 ± 6.18 | 7.90 ± 5.76 | 2.58 x10^-3^/0.312 | 0.168/ 0.968 |
| *Ruminococcus flavefaciens* | 0.08 ± 0.24 | 0.68 ± 2.06 | 0.16 ± 0.43 | 0.030/ 0.733 | 0.390/ 0.691 |
| *Alistipes purtredinis* | 0.75 ± 1.63 | 1.83 ± 2.41 | 1.88 ± 2.67 | 0.019/ 0.616 | 0.026/ 0.459 |
| *Bifidobacterium longum* | 2.80 ± 3.60 | 4.81 ± 6.64 | 6.94 ± 9.11 | 0.186/ 0.733 | 0.018/ 0.546 |
| *uncultured Oscillospira sp.* | 0.52 ± 0.82 | 0.81 ± 1.14 | 1.79 ± 3.11 | 0.091/ 0.733 | 0.006/ 0.295 |
| *Acidaminococcus intestine* | 1.33 ± 2.96 | 0.75 ± 2.19 | 0.44 ± 1.20 | 0.016/ 0.341 | 0.404/ 1.000 |
| *Megasphaera elsdenii* | 2.26 ± 4.16 | 0.56 ± 1.85 | 0.68 ± 2.41 | 0.003/ 0.312 | 0.783/ 1.000 |
| *Veillonella dispar* | 0.67 ± 1.22 | 0.29 ± 0.65 | 0.26 ± 1.02 | 0.010/ 0.423 | 0.378/ 1.000 |
| *Fusobacterium varium* | 1.10 ± 3.82 | 0.20 ± 0.79 | 0.04 ± 0.17 | 0.043/ 0.733 | 0.106/ 0.880 |
| *Collinsella aerofaciens* | 1.29 ± 0.25 | 1.00 ± 2.00 | 3.07 ± 5.52 | 0.824/ 0.921 | 9.18 x 10^-5^/ 0.034 |
| *Streptococcus thermophilus* | 0.42 ± 0.61 | 0.31 ± 0.42 | 0.89 ± 2.04 | 0.344/ 0.739 | 0.003/ 0.158 |

P values of P(-) vs. P(+)M(-) groups were calculated by Wilcoxon rank sum test. P values of P(+)M(-) vs. P(+)M(+) groups were calculated by Wilcoxon signed rank (paired) test.
