# Supplementary figures and images for: Impact of Enterobius vermicularis infection and mebendazole treatment on intestinal microbiota and host immune response
Source: PLoS Negl Trop Dis. 2017 Sep 25;11(9):e0005963. doi: 10.1371/journal.pntd.0005963 (PMC5629029; doi:10.1371/journal.pntd.0005963)

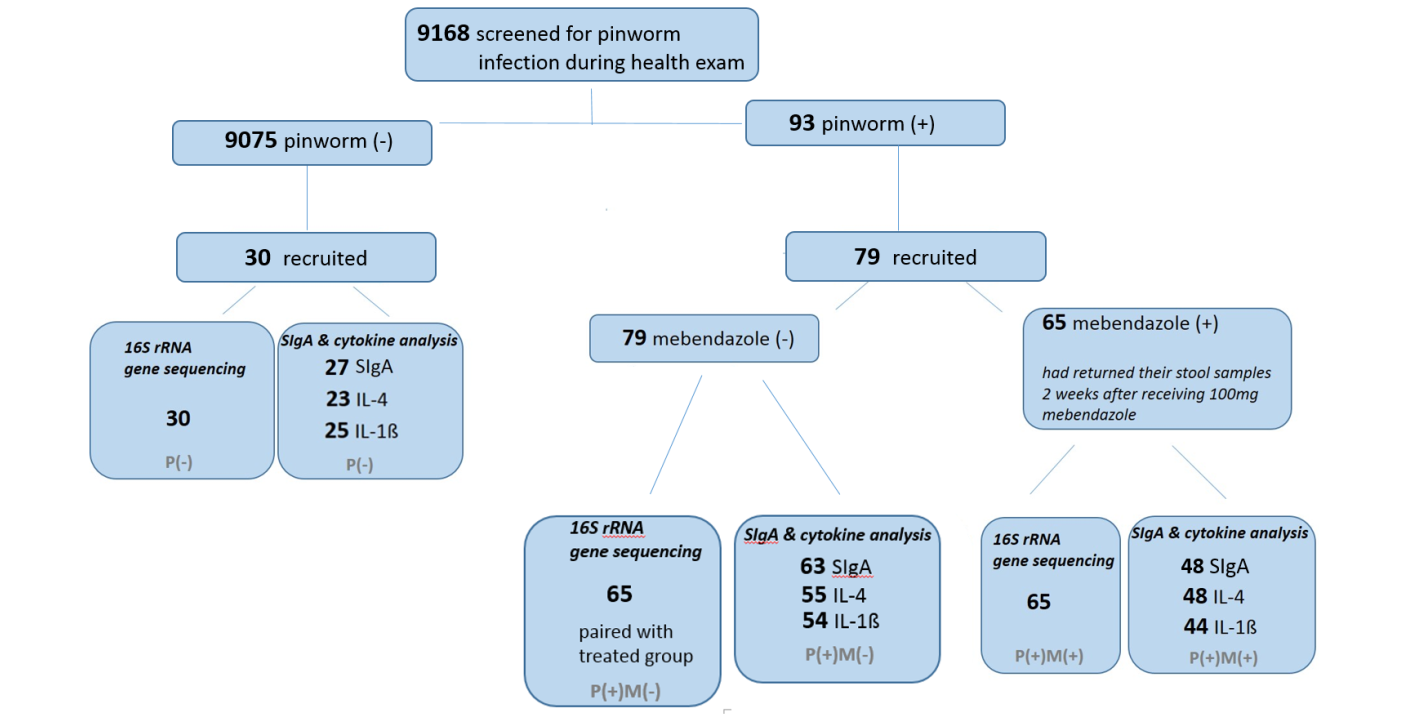

Supplement: S1 Fig — P(-): pinworm-negative; P(+)M(-): pinworm-infected, before mebendazole treatment; P(+)M(+): pinworm-infected, 2 weeks after mebendazole treatment. (TIF) [file pntd.0005963.s002.tif]

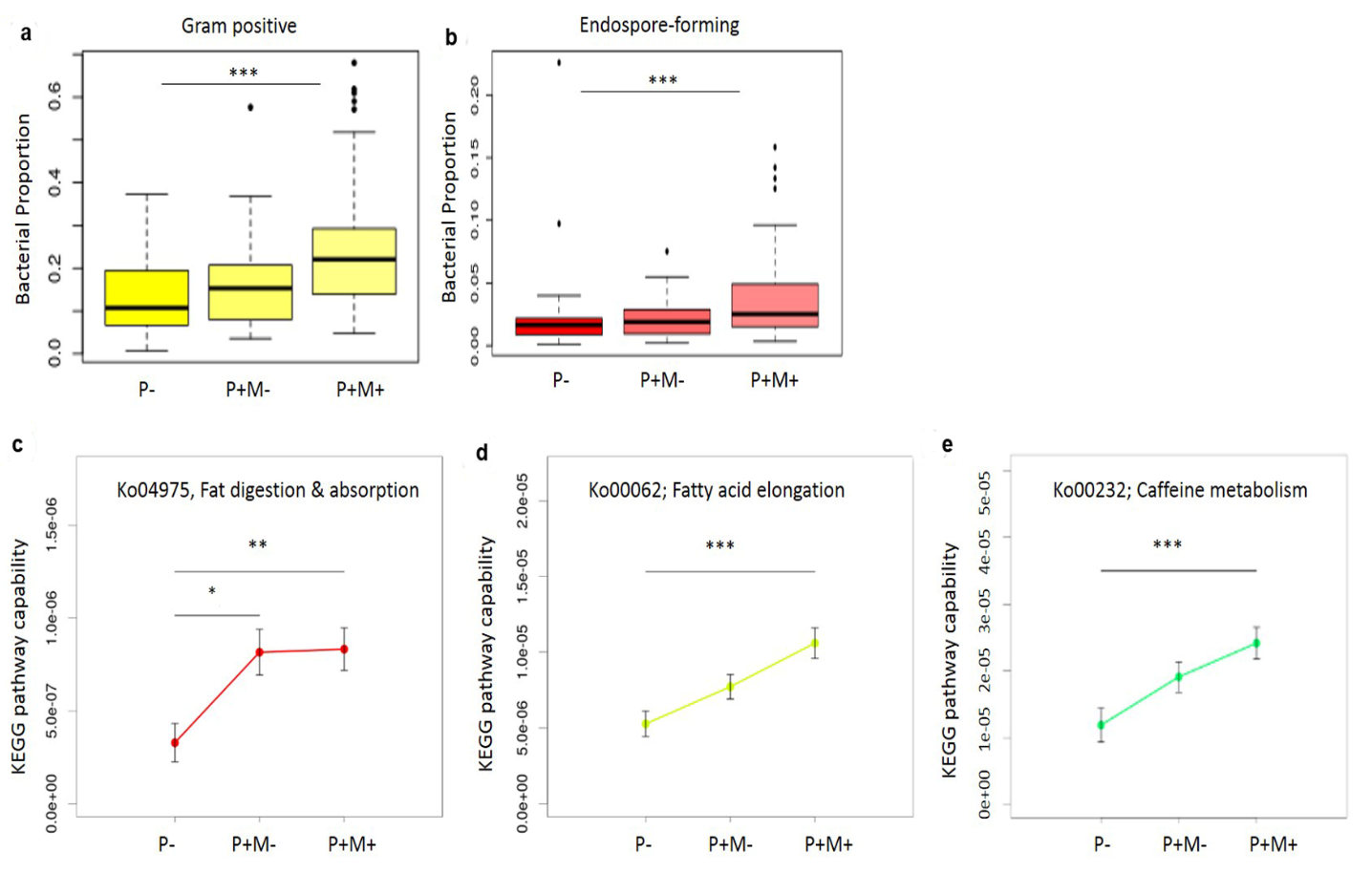

Supplement: S2 Fig — A-B. Box plots showing microbiology characteristic enrichment analyses based on Gram staining (a) and endospore-forming features (b). ***: p <0.001 by ANOVA test. c-e. Pathway enrichment analyses showing significant increase in fat metabolism (c), fatty acid elongation (d) and caffeine metabolism (e) pathways after pinworm infection plus mebendazole treatment. R package “Tax4Fun” was used to transform the OTU table into pathway activity value. ***: p <0.001, **: P<0.01, *: P<0.05 by ANOVA tests. (TIF) [file pntd.0005963.s003.tif]

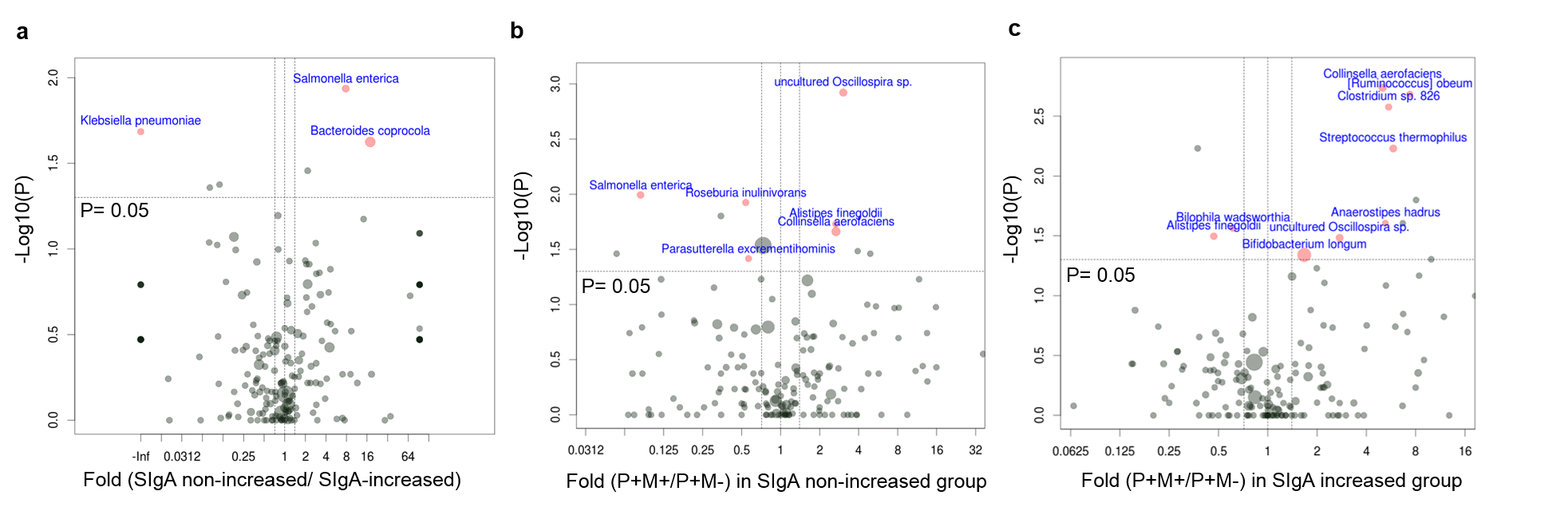

Supplement: S3 Fig — Volcano plots showing differentially expressed species-level bacteria comparing the untreated, pinworm-infected P+M- samples of SIgA non-increased group vs. the P+M- samples of SIgA-increased group (a), P+M+ samples vs. P+M- samples in SIgA non-increased group (b), and P+M+ samples vs. P+M- samples in SIgA-increased group (c). Red dots represent significant taxa analyzed by Wilcoxon tests. After p value corrections, those taxa remained to be significant (FDR< 0.05) are written in red, bacterial species with FDR> 0.05 are written in blue. Boundaries of significant fold change and p value are shown in dashed lines. Dot size represents average relative abundance. (TIF) [file pntd.0005963.s004.tif]
